# Current Performance of MALDI–TOF Mass Spectrometry Databases for the Identification of Dermatophyte Species

**DOI:** 10.3390/jof11050356

**Published:** 2025-05-05

**Authors:** David Rodriguez-Temporal, Daniel Adrados, Ana Alastruey-Izquierdo, Miriam Alkorta, Ana Candela, Andrés Canut, Carmen Castro, Carlos Gustavo Cilla, Juan de Dios Caballero, María Ercibengoa, Marina Fernández, Isabel Fradejas, Oscar Fraile, María José Goyanes, Ainhoa Gutiérrez, José Israel López, Concha López, Ana Isabel López-Calleja, Ramiro López-Medrano, Patricia Muñoz, Adriana María Ortega, Marina Oviaño, Javier Peman, María Rodríguez-Mayo, Alba Ruiz, Alexander Tristancho, Belén Rodríguez-Sánchez

**Affiliations:** 1 Clinical Microbiology and Infectious Diseases Department, Instituto de Investigación Sanitaria Gregorio Marañón, Hospital General Universitario Gregorio Marañón, 28007 Madrid, Spain; pmunoz@hggm.es (P.M.); mbelen.rodriguez@iisgm.com (B.R.-S.); 2 Microbiology Department, Complejo Hospitalario Insular-Las Palmas, 35016 Las Palmas de Gran Canaria, Spain; daniadr@gmail.com; 3 Mycology Reference Laboratory, National Center for Microbiology, Instituto de Salud Carlos III, 28222 Madrid, Spain; anaalastruey@isciii.es; 4 Clinical Microbiology Department, Hospital Universitario de Donostia, 20014 Donostia, Spain; miriam.alkortagurrutxaga@osakidetza.eus (M.A.); carlosgustavosantiago.cillaeguiluz@osakidetza.eus (C.G.C.); maria.ercibengoaarana@biodonostia.org (M.E.); 5 Clinical Microbiology Department, Hospital Universitario Lucus Augusti, 27003 Lugo, Spain; acandelagon@gmail.com; 6 Clinical Microbiology Department, Hospital Universitario de Álava, 01009 Álava, Spain; andres.canutblasco@osakidetza.eus (A.C.); marina.fernandeztorres@osakidetza.eus (M.F.); joseisrael.lopezmirones@osakidetza.eus (J.I.L.); 7 Microbiology Department, Hospital Universitario de Valme, 41014 Sevilla, Spain; carmencmendez@hotmail.com; 8 Microbiology Department, Hospital Universitario Ramón y Cajal, 28034 Madrid, Spain; juan.dcaballero@gmail.com; 9 Microbiology Department, Hospital Universitario Fundación Alcorcón, 28922 Alcorcón, Spain; isabel.fradejas@salud.madrid.org (I.F.); mariajose.goyanes@salud.madrid.org (M.J.G.); adrianamaria.ortega@salud.madrid.org (A.M.O.); 10 Microbiology Department, Complejo Asistencial de Ávila, 05071 Ávila, Spain; oscar.fraile.s@gmail.com; 11 Microbiology Department, Hospital Universitario La Princesa, 28006 Madrid, Spain; ainhoagutierrezcobos@gmail.com; 12 Microbiology Department, Hospital Universitario Miguel Servet, 50009 Zaragoza, Spain; clopezgo@salud.aragon.es (C.L.); alexander.tristancho1@gmail.com (A.T.); 13 Clinical Microbiology Department, Complejo Asistencial Universitario de León, 24008 León, Spain; rzlopez@saludcastillayleon.es; 14 CIBER de Enfermedades Respiratorias (CIBERES CB06/06/0058), 28029 Madrid, Spain; 15 Medicine Department, School of Medicine, Universidad Complutense de Madrid, 28040 Madrid, Spain; 16 Clinical Microbiology Department, Complejo Hospitalario Universitario A Coruña, 15006 A Coruña, Spain; marina.oviano.garcia@sergas.es (M.O.); maria.rodriguez.mayo@sergas.es (M.R.-M.); 17 Unidad de Micología Médica, Hospital Universitario La Fe, 46026 Valencia, Spain; javier.peman@gmail.com (J.P.); albacruiz@gmail.com (A.R.)

**Keywords:** dermatophytes, filamentous fungi, MALDI–TOF MS, identification

## Abstract

The identification of filamentous fungi by matrix-assisted laser desorption/ionization–time of flight mass spectrometry (MALDI–TOF MS) represents a challenge due to their complex taxonomy and the lack of comprehensive databases. The aim of this study was to evaluate the current status of available MALDI–TOF MS databases for the identification of dermatophytes, including commercial, in-house, and web-based databases. We collected 289 dermatophyte strains from different centers and analyzed them using four databases and a combination of them. The combination of commercial and in-house databases was shown to improve the identification rate and accuracy at the species level. For *Trichophyton rubrum*, the concordance among all databases was above 90.0%. For the *T. mentagrophytes* group, correct identification at the species level ranged from 30.0 to 78.9%, depending on the database, and showed very low agreement among them. The addition of the novel species *T. japonicum* to our in-house database resulted in the successful identification of this species. On the other hand, *T. interdigitale* and *T. tonsurans* were the species most frequently misidentified by MALDI–TOF MS. Through deep spectra analysis of both species, up to 29 protein peaks were found to be suitable for their differentiation, demonstrating the potential of peak analysis in differentiating closely related species. In conclusion, improvements of the databases with new strains resulted in increased identification accuracy at the species level. This, combined with peak analysis, could improve the overall identification of dermatophytes by MALDI–TOF MS in clinical laboratories.

## 1. Introduction

Dermatophytosis, or tinea, is one of the most common causes of superficial fungal infections in humans worldwide. The causative fungal species are characterized by their ability to degrade keratin present in the stratum corneum of the skin, hair, and nails of humans and animals [1,2]. As a result, dermatophytes are responsible for a wide spectrum of infections known as tinea corporis, tinea cruris, tinea pedis, tinea capitis and onychomycosis, classified according to the anatomical site affected. Their transmission mechanisms include direct contact with infected individuals or animals, or through contaminated soil, contributing to the persistence and spread of dermatophytosis in both community and healthcare settings [3]. Traditionally, dermatophytes have been classified into three main genera: *Trichophyton*, *Microsporum*, and *Epidermophyton*. However, advances in molecular taxonomy have led to the reclassification of several species, resulting in the description of additional genera such as *Nannizzia*, *Arthroderma*, *Lophophyton*, *Guarromyces*, and *Paraphyton* [4]. Despite these taxonomic changes, *Trichophyton* and *Microsporum* remain the most frequently implicated genera in human infections [5,6].

Accurate identification of dermatophyte species is crucial for epidemiological surveillance, therapeutic decision making, and the implementation of effective infection control measures, since different species exhibit variations in virulence and geographic distribution, underscoring the need for species-level identification in clinical practice [7]. Conventional identification methods for these fungi rely on fungal culture, microscopy, and morphological characterization [8]. While these techniques remain widely used, they present significant limitations, including the subjective interpretation of morphological traits, high variability among isolates, the need for extensive training, and being time-consuming. In addition, some dermatophyte species exhibit overlapping morphological characteristics, leading to frequent misidentifications. To overcome these challenges, molecular techniques based on DNA amplification and sequencing have been introduced for dermatophyte identification [9]. These methods provide more accurate identification, particularly in distinguishing closely related species and resolving taxonomic ambiguities. However, their routine application in clinical microbiology laboratories remains limited due to high costs, labor-intensive procedures, and the requirement for specialized equipment and personnel.

In recent years, matrix-assisted laser desorption ionization–time-of-flight mass spectrometry (MALDI–TOF MS) has emerged as a promising tool for fungal identification, offering rapid, accurate, and cost-effective species differentiation [10,11]. However, its application to dermatophyte identification presents significant challenges, mainly due to the complex taxonomy of this fungal group and the limited availability of robust reference databases, especially for the differentiation of closely related species [12,13,14]. For that reason, several studies have attempted to improve dermatophyte identification by creating their own in-house databases, often developed in single centers using local clinical isolates [15,16,17,18]. While these customized databases have demonstrated improved identification rates, they are often restricted to single-center studies and remain unavailable to the wider scientific community. Recently, efforts have been made to establish open-access databases to facilitate standardized fungal identification [19]. However, further validation of these databases is necessary to ensure their reliability across different geographic regions and laboratory settings.

The aim of this study was to evaluate current available MALDI–TOF MS databases, including our in-house database, for the identification of dermatophytes sourced from different centers. Additionally, we sought to explore species-specific protein markers to enhance the accuracy and potential of MALDI–TOF MS for discriminating between closely related species.

## 2. Materials and Methods

### 2.1. Fungal Strains

A total of 289 fungal strains were obtained from 14 different Spanish hospitals with preliminary morphological identification. After initial MALDI–TOF MS analysis, we confirmed that they encompassed, as follows: 187 strains of the *T. rubrum* group; 90 strains of the *T. mentagrophytes/benhamiae* group; 9 of the *Microsporum* genus; 2 of *Nannizzia*; and 1 of *E. flocossum*. All strains were subcultured on Sabouraud agar (Thermo Fisher, Waltham, MA, USA) and incubated at 35 °C for 5–14 days until visible growth was observed.

### 2.2. MALDI–TOF Mass Spectrometry

Using the sharp tip of an inoculating loop after removing the looped end, hyphae were collected from the external region of the colonies and resuspended in 300 µL of ultra-filtered water. Then, 900 µL of 100% ethanol was added and the tubes were vortexed for 10 min. The tubes were centrifuged at 13,000 rpm for 1 min, the supernatant was completely removed, and the pellet was allowed to dry at room temperature for 5 min. After that, protein extraction was performed by adding 20 µL of 70% formic acid and mixing with a micropipette. Then, 20 µL of acetonitrile was added; the mixture was homogenized with a micropipette and centrifuged at 13,000 rpm for 1 min. One microliter of the supernatant was deposited on the MALDI plate in triplicate, dried at room temperature, covered with α-hydroxycinnamic acid (HCCA) matrix, and allowed to dry again. The isolates were identified using the MBT Smart MALDI Biotyper (Bruker Daltonics, Bremen, Germany) with standard parameters and in the *Sample Type: Filamentous Fungi* acquisition mode. Score values of ≥1.8 and ≥1.6 were used as cutoffs for high- and low-confidence identification, respectively. Identifications below 1.6 were considered unreliable. The obtained spectra were compared using the following databases: the Bruker Filamentous Fungi Library 4.0 (FilFungi4.0); Bruker Filamentous Fungi, Version 2022 (FilFungi2022); an in-house fungal database (Hospital Gregorio Marañón, HGM); a combination of FilFungi2022 and HGM; and the online MSI platform with its fungal database (MSI; https://msi.happy-dev.fr/; accessed on 31 March 2025). For the latter, spectra were exported as .zip files from the MALDI Biotyper after each acquisition and uploaded to the MSI platform for identification. Although the latest version of the Bruker Filamentous Fungi database is from 2023, there are no changes compared to the 2022 version regarding the fungal groups and species included in this study.

### 2.3. Addition of New Reference Spectra to HGM Database

The strains used to expand the HGM database were analyzed as follows: they were deposited in 8 positions on the MALDI plate and each was measured 3 times, yielding up to 24 spectra per strain. The spectra were inspected using flexAnalysis 3.4 software (Bruker Daltonics) to detect and exclude any outliers or flat-line spectra. At least 20 high-quality spectra were selected to build a main spectrum profile (MSP) using MBT Compass Explorer software v4.0 (Bruker Daltonics). The expanded HGM database, containing 333 MSPs, has been deposited in the Zenodo repository (https://doi.org/10.5281/zenodo.15084054).

### 2.4. DNA Analysis

Isolates requiring molecular identification were subjected to DNA extraction. This involved incubating the fungi in 50 μL of lyticase and 200 μL of phosphate-buffered saline for 2 h at 37 °C, with agitation. Then, the tubes were centrifuged and the supernatant was discarded. After adding 20 μL of proteinase and 180 μL of lysis buffer, the samples were incubated overnight at 56 °C, with agitation. The following steps consisted of several washes and centrifugations following the manufacturer’s instructions (QIAamp DNA Mini Kit, Qiagen, Hilden, Germany) prior to PCR amplification of the internal transcribed spacer (ITS) region [20]. The procedure has been further described by Escribano et al. [21]. Briefly, the PCR products were purified and sequenced. The resulting sequences were identified by comparison with the reference database BLASTn from the National Biotechnology Center (NCBI, www.ncbi.nlm.nih.gov/BLAST) and by constructing phylogenetic trees using the neighbor-joining model with MEGA version 11.0 [22].

### 2.5. Protein Peak Analysis

Protein peak analysis of MALDI–TOF MS spectra, comparison of spectra, and the application of random forest analysis with k-fold cross-validation were performed using Clover Mass Spectrometry Data Analysis Software v1.12.1 (Clover MSDAS; Clover Biosoft, Granada, Spain). Peak selection was based on area-under-the-curve (AUC) values for each peak and by visual inspection.

### 2.6. Statistical Analysis

ANOVA analysis was applied to compare mean identification scores between databases; Fleiss’ kappa tests were used to assess concordance among the databases. Both tasks were carried out using R software v4.4.2 with the *tidyr* and *irr* packages [23]. The kappa value ranges from 0 to 1; the higher the value, the higher the concordance between the methods compared. The *t*-test for peak analysis, comparing mean peak intensities between different species, was performed using Clover MSDAS v1.12.1. A *p*-value of <0.05 was considered statistically significant.

## 3. Results

### 3.1. Comparison of Current Databases for T. rubrum Group

**Figure 1:**
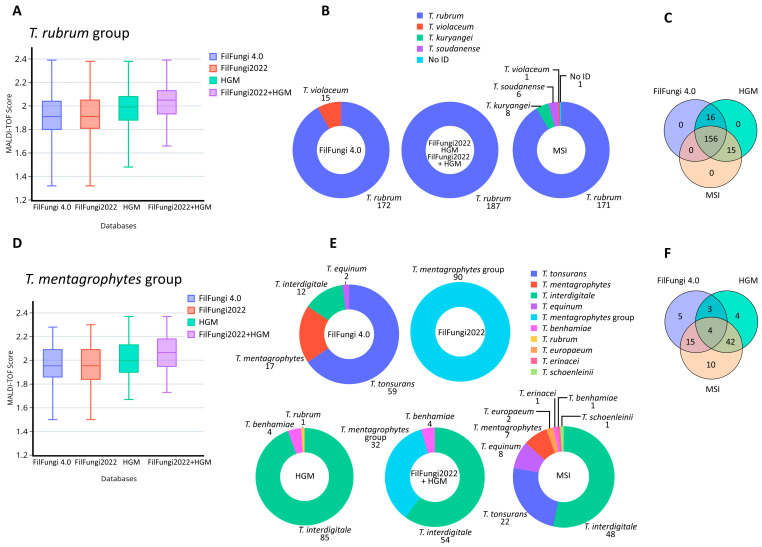
Identification of the most common groups studied (*T. rubrum* and *T. mentagrophytes* groups), comparing identification scores and classification results across different databases: (**A**) MALDI–TOF MS scores for *T. rubrum* group isolates obtained with each database, MSI not included since it uses different scoring system; (**B**) identification obtained with each database for *T. rubrum* group isolates; (**C**) concordance in species identification among FilFungi4.0, HGM and MSI databases for *T. rubrum* group isolates; (**D**) MALDI–TOF MS scores for *T. mentagrophytes* group isolates obtained with each database. MSI is not included since it uses a different scoring system; (**E**) identification obtained with each database for *T. mentagrophytes* group isolates; and (**F**) concordance in species identification among FilFungi4.0, HGM and MSI databases for *T. mentagrophytes* group isolates.

Among the 187 strains of the *T. rubrum* group analyzed, all were correctly identified at the group level by all databases used, with mean scores ranging from 1.9 to 2.0 (Figure 1A). Statistical analysis using ANOVA showed a significant value (*p* < 0.05) in the mean scores obtained with the combination of the FilFungi2022 + HGM databases compared to the others, as well as with the HGM database alone compared to the Bruker ones. No significant differences (*p* = 0.91) were observed between Bruker versions 4.0 and version 2022. Since identifications using the Bruker Filamentous Fungi Library Version 2022 are reported at the group level, and the HGM database contains only *T. rubrum* species within this group, the identification outcome was the same for all strains analyzed. Using the previous Bruker database (FilFungi4.0), 15 of the 187 *T. rubrum* group strains (8.0%) were misidentified as *T. violaceum* (Figure 1B; mean score 1.98), showing a 92.0% concordance with the other databases (Bruker version 2022 and HGM). An additional 16 isolates (8.6%) were identified as different species within the *T. rubrum* group by the MSI database (Figure 1B), which encompassed *T. kuryangei* (*n* = 8), *T. soudanense* (*n* = 6), *T. violaceum* (*n* = 1) and 1 not-identified isolate. MSI showed a concordance of 91.4% with the Bruker version 2022 and HGM databases. Overall, 156 (83.4%) *T. rubrum* isolates (Figure 1C) obtained the same identification from all evaluated databases. Strains with discordant results were definitively identified as *T. rubrum* through ITS region sequencing.

### 3.2. Comparison of Current Databases for T. mentagrophytes Group

Regarding the 90 strains of the *T. mentagrophytes* group (53 *T. interdigitale*, 25 *T. tonsurans*, 8 *T. mentagrophytes* and 4 *T. japonicum*; Table 1), all databases obtained mean scores between 1.96–2.06 (Figure 1D). ANOVA analysis showed a significant difference (*p* < 0.05) in the mean scores between the combined Bruker+HGM database and the Bruker databases alone (FilFungi4.0 and FilFungi2022). On the other hand, no significant differences were observed when comparing the Bruker+HGM database with the HGM alone (*p* = 0.170), the HGM database with the Bruker ones (*p* = 0.16), or between the Bruker commercial databases (*p* = 1.00). Due to updated spectrum names in FilFungi2022, all 90 isolates were uniformly classified as belonging to the *T. mentagrophytes* group by this database (Figure 1E).

For each of the evaluated databases, the number of correct identifications at the species level within the *T. mentagrophyes* group was as follows: 27 (30.0%) with FilFungi4.0; 53 (58.9%) with the HGM database; and 71 (78.9%) with the MSI database (Table 1). The concordance in species identification was 10.0% between FilFungi4.0 and HGM, 24.4% between MSI and FilFungi4.0, and 54.4% between HGM and MSI. The Fleiss’ kappa value was <0.0, indicating poor agreement among the three databases. Overall, four (4.4%) isolates obtained a correct species identification by all three databases (FilFungi4.0, HGM and MSI), all belonging to *T. interdigitale* (Figure 1F). The four *T. benhamiae* complex strains included in the study were identified as *T. benhamiae* by the HGM database (Table 1), while MSI classified these as *T. benhamiae* (*n* = 1), *T. europaeum* (*n* = 2) and *T. erinacei* (*n* = 1). ITS region sequencing confirmed that all four strains corresponded to *T. japonicum*. The later addition of two of these strains to the HGM database resulted in the correct identification at the species level of the remaining two strains.

### 3.3. Comparison of Current Databases for Other Genera

For the *Microsporum* genus (*n* = 9), the ANOVA analysis revealed no statistically significant differences (*p* = 0.69) in the identification scores among the databases. Regarding species identification, all were correctly classified by FilFungi2022 as part of the *M. audouinnii_canis* group. Among the other databases, six (66.6%) isolates were correctly identified by all. Identification at the species level was 100% using FilFungi4.0 and the MSI databases, whereas the HGM database identified 66.6% of isolates correctly, failing to identify *M. audouinnii* and misidentifying two *M. canis* isolates as *M. audouinnii*. Identification of discordant strains at the species level was confirmed by ITS sequencing.

Regarding the *Nannizzia* genus (*n* = 2), both Bruker and MSI databases correctly identified *N. incurvata*, while only the MSI database correctly identified *N. fulva*. The single *E. flocossum* isolate analyzed was correctly identified with a score of 1.8 by all databases except HGM, which does not contain this species.

### 3.4. Peak Analysis

**Figure 2:**
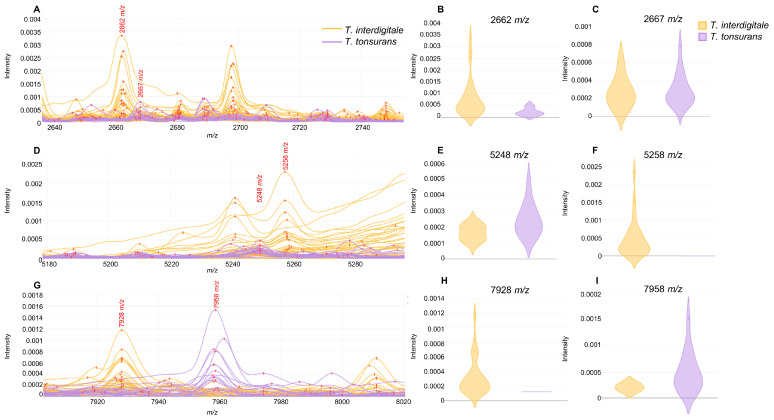
Most relevant peak pairs differentiating *T. interdigitale* and *T. tonsurans* isolates: (**A**) *m*/*z* region showing the shift between the 2662/2667 *m*/*z* peaks: (**B**) intensity plot of the 2662 *m*/*z* peak by species; (**C**) intensity plot of the 2667 *m*/*z* peak by species; (**D**) *m*/*z* region showing the shift between the 5248/5258 *m*/*z* peaks; (**E**) intensity plot of the 5248 *m*/*z* peak by species; (**F**) intensity plot of the 5258 *m*/*z* peak by species; (**G**) *m*/*z* region showing the shift of the 7928/7958 *m*/*z* peaks; (**H**) intensity plot of the 7928 *m*/*z* peak by species; and (**I**) intensity plot of the 7958 *m*/*z* peak by species.

For *T. interdigitale* and *T. tonsurans* species, considered the most common species within the *T. mentagrophytes* group, MALDI–TOF MS spectra were further analyzed to search species-specific marker peaks. The selection of peaks, based on their AUC values and visual inspection, resulted in 29 peaks, of which 23 showed statistical significance *p* < 0.05 according to the *t*-test (Table 2). The distribution of these peaks among species was as follows: 12 peaks predominated in *T. interdigitale* strains and 17 peaks predominated in *T. tonsurans* strains. Among all peaks, we found 3 pairs with very close *m*/*z* values, each corresponding to 1 species: the 2662/2667 *m*/*z* pair (Figure 2A), with 2662 *m*/*z* associated with *T. interdigitale* (Figure 2B) and 2667 *m*/*z* with *T. tonsurans* (Figure 2C); the 5248/5258 *m*/*z* pair (Figure 2D), with 5248 *m*/*z* for *T. tonsurans* (Figure 2E) and 5258 *m*/*z* for *T. interdigitale* (Figure 2F); and the 7928/7958 *m*/*z* pair (Figure 2G), with 7928 *m*/*z* for *T. interdigitale* (Figure 2H) and 7958 *m*/*z* for *T. tonsurans* (Figure 2I).

To evaluate the discriminatory power of these peaks, a random forest classification analysis was applied using the 29 peaks, applying a k-fold cross-validation. The optimized random forest hyperparameters resulted in 50 estimators, a maximum tree depth of 10, a maximum of 5 features per split, a minimum split size of 2 and a minimum of 1 sample per leaf. The k-fold cross-validation (k = 10) obtained an accuracy of 96.8%, with only two strains misclassified: one *T. interdigitale* misclassified as *T. tonsurans*, and one *T. tonsurans* strain misclassified as *T. interdigitale*.

## 4. Discussion

The rapid and reliable identification of dermatophytes in the microbiology laboratory is needed to rule out the presence of other clinically relevant filamentous fungi and to initiate adequate antifungal treatment. In recent years, MALDI–TOF MS has demonstrated its usefulness for the correct identification of fungal isolates, including dermatophytes [15,24,25,26]. However, the lack of robust databases still represents a challenge for the accurate identification of certain groups at the species level [27,28]. In addition, current databases, such as the Bruker commercial ones, have suffered important modifications in the latest updates, frequently grouping several species and reporting them at the group level rather than at the species level. On the other hand, open-access databases, such as the MSI, include a wide diversity of species, even some species currently under taxonomic revision. With the present study, we evaluated the current state of fungal databases for dermatophyte identification, expanding our in-house database with new species and further attempting the differentiation of difficult-to-identify species that are most frequently isolated such as *T. interdigitale* and *T. tonsurans*.

The *T. rubrum* group was shown to be accurately identified by all databases in a consistent manner. Regarding the scores obtained, the combination of commercial and in-house libraries significantly increased the scores, due to the higher number of spectra and better coverage of spectral variability from strains in our geographic area. Since this study did not include *T. violaceum,* the report at species and group levels was correct with the updated databases (FilFungi2022 and HGM, alone and combined). With the previous Bruker version (FilFungi4.0), some false-positive *T. violaceum* identifications were obtained (Figure 1B). The confusion by MALDI–TOF MS between these two species has also been observed before [15]. Using the MSI database, the presence of species names in this library that are not totally validated under the current taxonomy resulted in misclassification of a few strains such as *T. kuryangei*, *T. soudanense* and *T. violaceum*. Sequencing of the ITS region of these strains confirmed their identity as *T. rubrum* and highlights the need to use the current taxonomy in different databases. In fact, the species borderline of *T. kuryangei* is not clear; as a result, it is currently considered as a synonym of *T. rubrum* [9,21], although recent studies support it as a separate species [29].

Regarding the *T. mentagrophytes* group, as observed with the *T. rubrum* group, the identification scores increased significantly with the combined FilFungi2022+HGM database (Figure 1D). On the other hand, the *T. mentagrophytes* group obtained a high diversity of identification results when the databases were compared, which demonstrates the complexity and difficulty in accurately identifying these species by MALDI–TOF MS (Figure 1E). The updated Bruker database (FilFungi2022) encompassed both *T. mentagrophytes* and *T. benhamiae* complexes as a single group, resulting in the identification of all strains at the group level. With the previous database (FilFungi4.0), most identifications were assigned to *T. tonsurans* (Table 1). Conversely, the most common species identified by the HGM and MSI databases was *T. interdigitale*. In the first case, the HGM database did not contain *T. tonsurans*, which justifies the high proportion of *T. interdigitale* identifications. The MSI database, as previously observed, contains a high diversity of species, which resulted in greater taxonomic variability (Figure 1E). The fact that only 4.4% of the strains were consistently identified at the species level highlights the unequal construction of the databases for this group and its complexity. However, MSI was the most accurate database for the identification of the *T. mentagrophytes* group at the species level, with 78.9% correct identifications (Table 1). Overall, the most challenging species for MALDI–TOF MS were *T. tonsurans* and *T. interdigitale*. The misidentification of these two species, and of the *T. mentagrophytes* group in general, has been previously reported [24,30,31,32].

Interestingly, the four *T. japonicum* strains were only identified at the complex level by the HGM and MSI databases. These observations support the clear differentiation between *T. benhamiae* and *T. mentagrophytes* groups. The MSI database identified two of these as *T. europaeum* (Table 1). These two species were recently described as new components of the *T. benhamiae* group [14]; their differentiation remains challenging [33]. The correct identification of this species after the addition of some strains to the HGM database highlights the importance of enriching databases with more species and underlines the potential for identification at the species level in certain cases.

For the *Microsporum* genus, all databases yielded a similar performance regarding the scores, possibly due to the low number of strains analyzed. The updated Bruker database (FilFungi2022) combined *M. canis* and *M. audouinnii* as a group, although the set of strains used in this study was correctly identified at the species level by the previous database (FilFungi4.0). While the MSI database also correctly classified all isolates, the HGM database showed lower accuracy, which could be attributed to the limited representation of both species. Previous studies have reported confusion between these two species by both the Bruker and MSI databases [27,34].

Other genera analyzed in this study, such as *Nannizzia,* included few isolates and should be further studied. In our case, the lack of identification of *N. fulva* by most databases, except MSI, indicates the need for more comprehensive databases. Some isolates of this species have been previously analyzed by MALDI–TOF MS in some studies, with either no identification result or successful identification [15,17].

In our study, all identifications by ITS region sequencing were conclusive and unambiguous. Although this region is the most commonly used and considered the most discriminative for identification [29], some studies have indicated that additional genes, such as β-tubulin, may be necessary for a definitive identification of certain strains.

Beyond standard database-based identification, it was possible to find potential species-specific peaks for differentiating *T. interdigitale* and *T. tonsurans* through peak analysis (Table 2). Interestingly, we detected three peak pairs with very close *m*/*z* values, each corresponding to one of these species. Due to the close distance of peaks in each pair, we could speculate that they may correspond to the same protein, with slight modifications between the species. However, further proteomic analyses should be performed to better characterize these biomarkers. Despite the reported challenges in differentiating these species by MALDI–TOF MS databases due to their similarity, the identification process used by the MALDI Biotyper may contribute to their misidentification. This method uses only the region between 4000–10,000 *m*/*z* for peak selection and database comparison. In our analysis, we found 11 relevant peaks outside this range, which could improve identification accuracy. In fact, by applying a random forest classifier based on all detected peaks, it was possible to differentiate almost all strains of both species.

Peak analysis has not been common in previous studies using MALDI–TOF MS for dermatophytes. Cmoková et. al. performed a similar analysis with members of the *T. benhamiae* group, detecting variable spectra regions specific to the species analyzed [14]. However, manual inspection of spectra may lead to the loss of relevant information. For that reason, we combined visual inspection with automated peak detection in order to identify differences between the species studied. In addition, the use of machine learning methods, such as random forest, offers an alternative method for the in-depth exploration of spectra, enabling the simultaneous analysis of large datasets. These methods have already proven useful in bacterial studies [35], although they are still less commonly applied to fungi [36].

This study presents some limitations. First, some species were not isolated by the participating centers; therefore, they could not be evaluated, although they are less commonly isolated in our geographic area. Specifically, *T. indotineae*, a species of increasing interest due to its intrinsic resistance to terbinafine, was not evaluated; more importantly, it is still not included in commercial databases [37]. Second, only our in-house database (HGM) and the MSI platform were evaluated. Further studies are needed to develop a comprehensive database that includes all dermatophyte species and to validate it using a broad and diverse collection of strains from different origins in order to assess the full potential of MALDI–TOF MS-based identification.

## 5. Conclusions

The taxonomy of dermatophytes is complex; species boundaries within some groups are still under debate. For the accurate identification of these fungi, MALDI–TOF MS databases must not only be comprehensive in terms of species coverage but also aligned with the current taxonomic standards. This study has shown that the creation of in-house databases and the inclusion of additional species enable precise species-level identification. However, there remains a pressing need to establish universal and comprehensive databases accessible to MALDI–TOF MS users in clinical laboratories. In addition, novel strategies, such as the deep analysis of spectra, showed that species-specific differences exist and that their differentiation can be enabled through MALDI–TOF MS.

## Figures and Tables

**Table 1:** Number of isolates from each species within *T. mentagrophytes* group and the classification result obtained by each single database used. Values in bold represent the correctly identified isolates at the species level.

Database	Species Result	***T. interdigitale*** (***n*** = 53)	***T. tonsurans*** (***n*** = 25)	***T. mentagrophytes*** (***n*** = 8)	***T. japonicum*** (***n*** = 4)
FilFungi4.0	*T. tonsurans*	36	**18**	5	-
	*T. mentagrophytes*	10	4	**2**	1
	*T. interdigitale*	**7**	1	1	3
	*T. equinum*	-	2	-	-
HGM	*T. interdigitale*	**53**	25	7	-
	*T. benhamiae*	-	-	-	4
	*T. rubrum*	-	-	1	-
MSI	*T. interdigitale*	**46**	-	2	-
	*T. tonsurans*	3	**19**	-	-
	*T. equinum*	3	5	-	-
	*T. mentagrophytes*	1	-	**6**	-
	*T. europaeum*	-	-	-	2
	*T. erinacei*	-	-	-	1
	*T. benhamiae*	-	-	-	1
	*T. schoenleinii*	-	1	-	-

**Table 2:** The most relevant peaks for the differentiation between *T. interdigitale* and *T. tonsurans* species, based on AUC values and the *t*-test. The peak list was obtained using Clover Mass Spectrometry Data Analysis Software v1.12.1.

Peaks (***m/z***)	AUC	Species	***t***-Test ***p*** Value
2512	0.9021	*T. tonsurans*	**<0.05**
2662	0.8256	*T. interdigitale*	**<0.05**
2667	0.9045	*T. tonsurans*	**<0.05**
2747	0.8629	*T. interdigitale*	**<0.05**
2887	0.8213	*T. interdigitale*	**<0.05**
3630	0.8568	*T. tonsurans*	**<0.05**
3740	0.6860	*T. interdigitale*	>0.05
4664	0.8397	*T. tonsurans*	**<0.05**
4749	0.8152	*T. tonsurans*	>0.05
5248	0.7332	*T. tonsurans*	**<0.05**
5258	0.8488	*T. interdigitale*	**<0.05**
5329	0.8960	*T. interdigitale*	**<0.05**
5400	0.8531	*T. interdigitale*	**<0.05**
5629	0.8299	*T. tonsurans*	**<0.05**
5852	0.8042	*T. interdigitale*	**<0.05**
6357	0.8237	*T. tonsurans*	**<0.05**
6458	0.7858	*T. interdigitale*	**<0.05**
6533	0.8409	*T. tonsurans*	**<0.05**
7304	0.8017	*T. tonsurans*	**<0.05**
7327	0.8042	*T. tonsurans*	**<0.05**
7865	0.8384	*T. tonsurans*	>0.05
7928	0.8739	*T. interdigitale*	**<0.05**
7958	0.9088	*T. tonsurans*	**<0.05**
9367	0.8244	*T. tonsurans*	**<0.05**
9499	0.8568	*T. tonsurans*	**<0.05**
11,228	0.8397	*T. interdigitale*	**<0.05**
12,251	0.8684	*T. tonsurans*	>0.05
12,419	0.7607	*T. interdigitale*	>0.05
12,435	0.8403	*T. tonsurans*	>0.05

AUC: area under the curve.

## Data Availability

Data used in this study are available upon reasonable request.
